# Prevention of sudden unexpected postnatal collapse in wellbeing newborns by remote digital health technologies

**DOI:** 10.3389/fdgth.2025.1598541

**Published:** 2025-09-19

**Authors:** Massimo Berger, Adalberto Brach del Prever, Michele Mario Calvo, Roberto Bellino, Davide Gallina, Fabio Stefano Timeus, Fabrizio Bogliatto

**Affiliations:** ^1^ Pediatrics and Neonatology, PO Ivrea, Azienda Sanitaria Locale Torino 4 (ASLTO4), Ivrea, Torino, Italy; ^2^ Pediatrics and Neonatology, PO Ciriè, Azienda Sanitaria Locale Torino 4 (ASLTO4), Ciriè, Torino, Italy; ^3^ Pediatrics and Neonatology, PO Chivasso, Azienda Sanitaria Locale Torino 4 (ASLTO4), Chivasso, Torino, Italy; ^4^ Gynecology and Obstetrics, PO Ciriè, Azienda Sanitaria Locale Torino 4 (ASLTO4), Ciriè, Torino, Italy; ^5^ Gynecology and Obstetrics, PO Chivasso, Azienda Sanitaria Locale Torino 4 (ASLTO4), Chivasso, Torino, Italy; ^6^ Department of Head of the Maternal and Child, Gynecology and Obstetrics, PO Ivrea, Azienda Sanitaria Locale Torino 4 (ASLTO4), Ivrea, Torino, Italy

**Keywords:** sudden unexpected postnatal collapse, early skin-to-skin contact, wireless monitoring, SUPC, neonate, cardiopulmonary monitoring, early skin to skin

## Abstract

**Introduction:**

To prevent the Sudden Unexpected Postnatal Collapse (SUPC) this approach was carried out. SUPC is a rare and devastating event for the child and their family. Currently, no diagnostic prediction model is available to calculate the individual newborn risk.

**Patient and methods:**

To prevent SUPC, the Department of Maternal and Child Health at ASLTO4 in Piedmont, Northern Italy, has implemented wireless cardiopulmonary monitoring for all newborns during the first 24 h of life, starting on June 10th, 2023, to December 31st, 2024. The study involved approximately 2,000 newborns from three Spoke hospitals in Northern Italy. The aim of the study was to evaluate the feasibility of wireless monitoring in a large series of newborns.

**Results:**

On more than 2,000 newborns, we have seen parental refusal in only two cases. The system was well accepted by the families after adequate explanation of the monitoring modalities and its meaning. The wireless system has in no way hindered the skin-to-skin moment nor delayed the time of attachment to the breast and the usual neonatal screening procedures. The introduction of this new technology has brought increased serenity to parents, especially in situations of severe tiredness after troubled births or after cesarean delivery. As a very preliminary results in 2,250 newborns the monitoring system detected various pathological events, in particular two cases of SUPC which were promptly resuscitated without subsequent neurological sequelae.

**Conclusions:**

We report on our proof-of-concept innovative digital approach to intercept SUPCs as soon as possible. Through this study we want to demonstrate that it is possible to carry out large-scale multicenter monitoring, without interfering with breast attachment and the initial mother-infant relationship. The limitations of the study mainly concern the fact that this monitoring was carried out on term or late pre-term infants. This was due to the unavailability of a neonatal intensive care (TIN) within our hospitals and therefore severe preterm children were born or transferred early to a third level hospital.

## Introduction

Early skin-to-skin contact (ESSC) in the first hours postpartum provides clear benefits for both the mother and the newborn and is now a generalized practice (1–3). The newborn's position and contact during ESSC includes being placed in a prone position on the maternal abdomen/chest, with its face turned sideways and its respiratory tract unrestricted [Task Force SUPC-TIN (Indicazioni sulla prevenzione e gestione del Collasso Postnatale SUPC)] (4). In this position, the baby, once dried, must be protected from cold heat with a dry sheet and a cover. In some cases, a cap can be placed on the dried head to improve thermal control. During ESSC, the mother should be positioned at a 45° semi-reclining angle.

Although deemed suitable for ESSC, the newborn baby must be carefully and continuously monitored by delivery room personnel in the first 10–15 min of life, with ESSC continuing for 2 h.

According to the British Association of Perinatal Medicine, SUPC is defined as any newborn with gestational age (GA) >35 weeks, APGAR score (5, 6) at 5 min >8, and assessed as suitable for standard neonatal care, who presents sudden cardiovascular and respiratory impairment in the first week of life. SUPC requires prompt intervention with resuscitation procedures using intermittent positive pressure ventilation, but it can result in intensive neonatal care, encephalopathy, or death (6, 7). Since SUPC events have multifactorial pathogenesis, a protective effect cannot be attributed to clinical surveillance alone, even if it is regular.

There has been a rise in the reporting of SUPC cases during ESSC. In 2017, the World Health Organization calculated that 1.6–5 cases per 100,000 live births occur in the first 2 h after delivery. Additionally, 60% of babies with SUPS died during ESSC, and up to 50% of those who survived had neurological sequelae. In their regional and population-based study, Polberger and Svenningsen reported that infants considered healthy at birth can experience SUPC between 6 and 100 h after birth (7).

The etiology of SUPC is unknown, and similarly to sudden infant death syndrome (SIDS), studies on defining the causes of SUPC are difficult to conduct (8, 9). Additionally, oxygen desaturation episodes during ESSC within the first 2 h after delivery are extremely common in healthy full- term newborns (10). Although the risks of SUPC have not been estimated, the following related factors have been identified: primiparous mothers, mothers with a body mass index (BMI) > 25 kg/m^2^, mothers who have used medications that cause drowsiness or sedation, tiredness, and sleepiness after childbirth; newborns placed in a prone position over the mother's body, breastfeeding, absence of surveillance by a companion or health personnel, and the mother being distracted by electronic devices (mobile phones, etc.) particularly during ESSC (8, 9). A very recent paper by Colvin JD documented that “compared with SUID at older ages, SUPC was associated with older and primiparous mothers, swaddling, and the caregiver falling asleep while feeding the infant.” Their conclusion was to reinforce safe sleep recommendations and provide guidance regarding situations when parents may fall asleep during a feeding (11). Another paper had also documented how it was possible to reduce the number of SUPCs through monitoring of oxygen saturation during skin-to-skin care (12).

The aim of this study is to demonstrate how it is possible to monitor non-invasively, even in the first hours of life, cardio-respiratory parameters in a large population of newborns. Monitoring, made available by wireless technology, has proven to be effective and non-invasive in all patients who accepted the study. The incidence but especially the outcomes of any SUPCs, will be analyzed in a future comparative analysis between patients wirelessly monitored and those not monitored in a previous timeframe.

The study was conducted in three Spoke hospitals in Northern Italy (Piedmont, ASLTO4). This area has been home to strong immigration since the 60s from Southern Italy and subsequently, in much more recent years, from North Africa and recently from Eastern Europe.

## Burden of clinical assessment

Recent national surveys from Germany and the UK have used different criteria for inclusion: SUPC is defined as occurring within the first 24 postnatal hours, excluding (in Germany) and including (in the UK) identified and possibly preventable causes (9). Both national surveys report an incidence of about 3 cases per 100,000 infants. However, recently published guidelines for the investigation of SUPC include infants with sudden unexpected collapse during the first week of life. These “Guidelines for the investigation of newborn infants who suffer a sudden and unexpected postnatal collapse in the first week of life” mention several published reports concerning SUPC (13, 14).

It is important to be cautious when comparing estimates of incidence when reports use different inclusion and exclusion criteria for SUPC. Published reports have different definitions on when unexpected collapses occur in seemingly healthy infants (between 2 h and within 7 days after birth) and when gestational age is >35 or >38 weeks. There are numerous reports of cases from French maternity wards, with estimates estimating the incidences to be 3.2–3.6 per 100,000. According to Spain's reports, the incidence rate is between 5.5 and 7.4 per 100,000. However, these French and Spanish studies have only included cases occurring within 2 h after birth.

Recent national surveys from the UK and Germany have used yet another time criterion for inclusion; SUPC is defined as occurring within the first 24 postnatal hours. Both national surveys report an incidence of about 3 cases per 100,000 infants. Recently, guidelines for the investigation of SUPC have included infants who collapsed unexpectedly during the first postnatal week. Moreover, in the reports that include infants of postnatal age <3 or <7 days, roughly half of SUPC cases reported occur after 24 h. If reports exclude SUPC cases that happen after 24 h, the incidence of sudden unexpected postnatal collapses in presumably healthy babies (14–21) will be underestimated.

Most publications and authors indicate that the number of reported SUPC cases is lower than what occurs in the wards and only reflects the most critical events. Events with rapid and favorable outcomes could easily be missed in large surveys. One recent retrospective study indicates that the prevalence of early sudden unexpected death in infancy **(**SUDI) has not changed in northwest England over 35 years and is considered rare [3.5 SUDI per 100,000 (22)]. In contrast to the UK survey and report, Rodriguez-Alarcon et al., in their regional study, show a significant increase in SUPC since December 2008 (23). They associate the increased SUPC incidence with altered routines in maternity wards and the encouragement of early skin-to-skin contact without adequate surveillance. In three Stockholm University Hospitals, which examined some 68,000 births over 2.5 years between January 2010 and July 2012, the incidence of SUPC was about half of that reported in the recent Spanish report, but it is nearly ten times higher than in the national surveys in the UK and Germany (9).

Most SUPC events happen within 2 h of birth, often during the initial breastfeeding attempt. Prone position, skin-to-skin contact, and co-bedding are all linked to SUPC. According to Poets et al., who examined 31 cases and 93 controls, there is a 6.4 odds ratio for SUPC to occur in a prone or potentially asphyxiating position when an identifiable cause is not known (24).

In brief, the risk factors for SUPC include a prone position, first breastfeeding attempt, co-bedding, primiparous mother, and being left alone with the baby during the first hours after birth.

Adequate information is an essential prerequisite for the prediction of SUPC. Generally, the advice given includes: parental education to use the recommended supine position and to avoid recognized risk factors for SUPC (co-bedding or bed-sharing, prone sleeping on soft bedding, face down and heat covering), appropriate non-invasive surveillance by caregivers aware of the possibility of SUPC and, if possible, continuous supervision of mothers at risk (primiparous, alone and exhausted), medical supervision and discussion about ESSC in the case of pathological conditions of the mother or the newborn, and positioning the infant supine to avoid mechanical airway obstruction or a possible asphyxiating risk.

## The need for digitaltechnology

By developing and integrating wearable sensing technologies with wireless communication techniques and data processing algorithms, continuous monitoring of neonates' vital signs and physiological parameters can be accomplished. Clinicians can be provided with remote data by a wearable sensor system as a clinical tool. Traditional sensors and medical instruments are not suitable for wearable physiological monitoring applications due to their unsuitability for long-term wear and discomfort caused by skin irritation, entanglement in wires, and sleep interruption.

While cardiorespiratory monitoring has already been introduced in non-European countries, experiences in Europe are very limited. A similar postnatal monitoring experience has already been evaluated in Italy using the system called “NewMoon—Smart Garments in Newborns and Babies Monitoring.” This project, coordinated by the “Fondazione IRCCS CA Granda Ospedale Maggiore Policlinico—Mangiagalli—Italy,” involved exclusive monitoring for the first 2–4 h of life as part of the European Horizon 2020 project from May 1, 2015, to November 30, 2015.

Within the Maternal and Child Department of ASLTO4, located in Piedmont, Northern Italy, new technology was acquired to improve the quality of care for newborns and their families. The project, conceived in the summer of 2022, was implemented in the Clinic in June 2023 and has allowed the monitoring of about 2000 newborns during the first 24 h of life at the hospitals of Ciriè, Chivasso, and Ivrea (ASLTO4, Northern Italy). The monitoring started on 10th June 2023 to December 31st, 2024.

The aim of this study is to demonstrate how it is possible to monitor non-invasively, even in the first hours of life, cardio-respiratory parameters in a large population of newborns. Monitoring, made available by wireless technology, has proven to be effective and non-invasive in all patients who accepted the study. The incidence but especially the outcomes of any SUPCs will be analyzed in a future comparative analysis between patients monitored and those not monitored in a previous timeframe.

All newborns were subjected to wireless monitoring for the first 24 h of life, regardless of whether they were born at term or preterm. At the Ivrea and Chivasso hospitals, babies are born starting from the 34th week of pregnancy, while at the Ciriè hospital starting from the 32nd week of pregnancy. The only exclusion criterion was parental refusal.

It did not require approval from the ethics committee since monitoring of heart rate, oxygen saturation and cardiac perfusion index are part of the clinical routine for the screening of congenital heart disease.

The protocol involves several phases:

1. An initial phase of explaining SUPC to parents or legal guardians and obtaining relative consent for monitoring during the pre-delivery course.
2. Positioning the wireless sensor at the foot of the newborn during the ESSC phase (two hours) and for the next 22 h. The Radius PPG single-use sensor consists of a very light sensor with an adjustable wrist strap and has a battery life of 96 h.
3. Wireless connection of the sensor to a host device located outside the delivery room and the mother's hospital room, ensuring it does not interfere with common practices of the second parent's approach to the newborn and care. This device, equipped with visual and audible alarms, is set to detect significant bradycardia rather than desaturation, regardless of the movements of the newborn and the mother. Remote monitoring *via* a central unit capable of recording up to 500 devices.
4. Recording, storing, and possibly downloading the monitoring data of individual newborns later.

Each newborn was monitored from the ESSC to 24 h later, after an appropriate explanation to parents. The parameters were set as follows:

Using the Patient SafetyNet Notification/Message to Physician mode, users can define clinical activities for the notification devices. The system is designed to send alarm/alert information from the instrument to a doctor's wearable pager, IP device, or portable device within approximately ten seconds under normal load conditions. The system can support up to 50 Patient SafetyNet display stations and up to 500 Po C devices.

The digital system will therefore allow us to record and signal digitally and acoustically, changes in severe and not severe cardio-respiratory parameters and to allow immediately the start of the necessary resuscitation maneuvers (Table 1).

**Table 1:** Vital parameters monitored by wireless sensors.

Vital parameters	By the company	Modified to
SpO_2_ upper limit	OFF	OFF
SpO_2_ lower limit	88	85
HR upper limit	200	220
HR lower limit	80	80
Pi upper limit	OFF	OFF
Pi lower limit	0.3	0.2

SpO_2_: Blood saturation is the ratio of oxygen in the blood to the maximum amount of oxygen that can be carried by the blood. Normal blood saturation values range between 95% and 100%.

HR: Heart rate (HR) is the speed of contractions or heartbeats, measured by the number of beats per minute (bpm).

**Figure 1:**
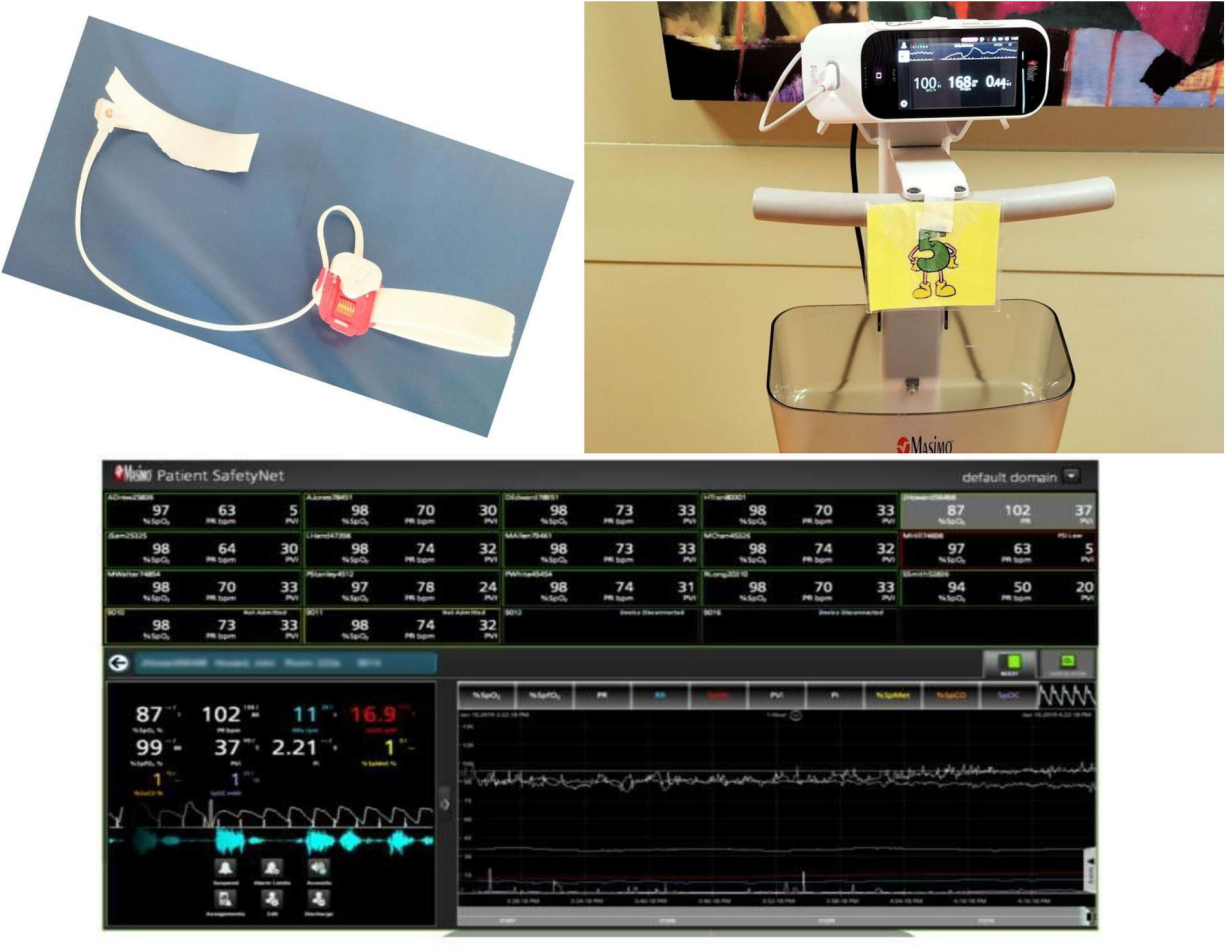
Newborn pulse oximetry sensor, device positioned outside the mother's room and central monitoring station. Screenshot from: Patient SafetyNet (https://www.masimo.co.uk/).

Pi: Perfusion index (Pi) is a numerical value indicating the intensity of the pulse at the point where the sensor is placed. This is a relative value that varies from patient to patient and depending on the area where the sensor is placed (Figure 1).

## Results

The aim of this study was to evaluate the feasibility of wireless cardiopulmonary monitoring in a large population of newborns from different ethnic groups. We observed almost total adherence to the protocol with only two families who preferred not to participate in the study for unclear reasons.

We report that our innovative wireless approach can be performed in newborns also in a Spoke Hospital. This study is intended as a proof of concept. The comparison with other monitoring methods (nursing, midwifery, partner or other family members) of the puerperia is not the aim of the study. In other words, the authors are conducting a second analysis of the results with comparison with historical monitoring (monitoring by nurses, obstetricians, doctors but also by the partner or other family members).

The monitoring allowed us to observe, for the first 24 h of life, the heart rate, oxygen saturation and perfusion index in over 2,000 newborns. The monitoring did not interfere with the skin-to-skin period, nor with the attachment of the newborn to the mother's breast, nor with any other clinical practice necessary on the newborn.

As preliminary results, out of 2,225 infants in which monitoring was applied, a total of 40 pathological events were detected by alerting the system with audible and visual alarms.

Among these we had two cases of potential SUPC that allowed the rapid intervention and resuscitation of the newborn:

- In a first case, the monitoring system detected desaturation during the rooming-in; at the timely arrival of the medical-nursing team the newborn (male, third born, born by spontaneous vaginal delivery and eventually without apparent risk factors) was in the mother's bed, who had fallen asleep during feeding in a suffocating position for the newborn (covering with the breast of the baby's face). Moreover, the parents had also applied a cap with a tight strap on the neck; there were no caregiver. The newborn was rescued by the physician on duty: the cap was removed, with rapid resolution of perioral cyanosis and immediate normalization of vital parameters.
- In the second case it involved a late preterm newborn born at 36 weeks of gestational age from vaginal delivery: APGAR 9/9, adeguate for gestational age (AGA), with a premature rupture of the amniotic membranes (PROM) of about 18 h before delivery, rectal-vaginal swab result unknown. At 20 h of life, the monitoring system detected desaturation. Upon the arrival of the nurse in the room, the baby was completely under the blankets with cyanosis on the right side decubitus face against the body of the mother, who had fallen asleep. Staff performed tactile stimulation and free-flow oxygen administration with prompt recovery.

In addition, 3 other cases of desaturation were detected, which led to the detection of:

- A case of early onset sepsis (EOS) in a full-term baby born not observation for infectious risk as PROM >18 h.
- A case of cerebral hemorrhage in an infant without apparent risk factors that presented repeated transient desaturations up to SatO2 60%–70% with spontaneous resolution.
- A case that has presented in the first days of life episodes of desaturation without known cause with spontaneous resolution.

Finally, 35 cases of neonatal bradycardia were detected. In 5 cases they were finally diagnosed as early onset neonatal sepsis (EOS) and promptly treated in accordance with the national protocols (SIN, Società Italiana di Neonatologia).

## Conclusions

We report our proof-of-concept innovative digital approach to intercept SUPCs as soon as possible. All parents or legal guardians except two have agreed to the vital sign monitoring according to the protocol. It did not have any impact on the early mother-child interaction but, rather, it guaranteed serenity and the possibility of rest. The results will be measured through the digital recording of severe and non-severe cardiorespiratory events in patients born in the hospitals of Ciriè, Chivasso and Ivrea (ASLTO4), Northern Italy.

The limits of the study include the costs of the equipment, especially the wireless sensors, the commitment of the nursing and obstetric staff to intervene frequently when the sensor signals a desaturation or bradycardia (often transitory and linked to the movement of the newborn) but also the need to dedicate time to explain to all parents the purposes of the monitoring. This last aspect can be particularly relevant in centers with a high number of newborns. Another limit of the study mainly concern the fact that this monitoring was carried out on term or late preterm infants. This was due to the unavailability of a neonatal intensive care (TIN) within our hospitals and therefore severe preterm children were born or transferred early to third level hospital.

The next step of this large multicenter hospital-based study will be, after having demonstrated the feasibility of the study in newborns in three Spoke hospitals for the first 24 h of life, to describe the cardio-pulmonary events, but also hemorrhagic or epileptic ones, intercepted through wireless monitoring.

## Data Availability

The raw data supporting the conclusions of this article will be made available by the authors, without undue reservation.
